# The politicians’ perspectives on participation in mammographic screening: an interview-based study from a region in Sweden

**DOI:** 10.1186/s13690-021-00576-6

**Published:** 2021-04-17

**Authors:** Maria Norfjord van Zyl, Per Tillgren, Margareta Asp

**Affiliations:** 1 Division of Public Health Sciences, School of Health, Care and Social Welfare, Mälardalen University, Box 883, 721 23 Västerås, Sweden; 2 Division of Caring Sciences and Health Care Pedagogics, School of Health, Care and Social Welfare, Mälardalen University, Box 883, 721 23 Västerås, Sweden

**Keywords:** Decision making, Mammographic screening, Participation, Politicians, Prioritisation, Public health, Social determinants

## Abstract

**Background:**

Breast cancer is the most common cancer type among women globally. To facilitate early detection, all 40–74-year-old female residents of Sweden are invited to participate in a population-based mammographic screening programme. Approximately 20% of all invited women decline the offer, and if this is due to systematic differences that can be adjusted, it can indicate inequity in healthcare. Assessment of and being updated about the health and healthcare of the residents are largely the responsibilities of the self-governed regions in Sweden. The understanding of the residents’ health serves as a basis for decision making and priority setting. This study aims to describe how politicians representing a region in Sweden perceive women’s participation in mammographic screening and the politicians’ own possibility to promote such participation.

**Methods:**

Qualitative thematic analysis was conducted on the data obtained from individual semi-structured interviews held in 2019. The interviewees comprised ten politicians (six women and four men, 38–71 years old) representing a sub-committee focusing on public health and healthcare issues.

**Results:**

Two main themes have been identified: 1) expected actions and 2) expected conditions for acting, including a total of four sub-themes. According to the politicians, the expected actions, such as obtaining information and being updated about matters regarding mammographic screening, concern both the women invited to the screening and the politicians themselves. Additionally, for both the individual and the healthcare organisation, here represented by the politicians, expected actions entail a shared commitment to maintain health. The expected conditions for acting refer to the politician’s awareness of the factors influencing the women’s decision to undergo or refuse the screening and having the resources to enable taking actions to facilitate participation.

**Conclusions:**

Expected actions and expected conditions for acting are tightly connected and entail some form of prioritisation by the politicians. Setting the priorities can be based on information about the purpose of the screening and an understanding of social determinants’ impacts on women’s decision to refrain from mammographic screening, as well as available resources.

**Supplementary Information:**

The online version contains supplementary material available at 10.1186/s13690-021-00576-6.

## Background

Breast cancer is the leading type of cancer among women globally; in 2018, the incidence of breast cancer was 2,088,849 [1]. Predictions estimate that in 2040, the global incidence of women with a breast cancer diagnosis will be approximately 3,000,000 cases, and for Sweden, approximately 8000–9400 cases are expected, equivalent to a 17.1% increase from 2018 [2]. One method of early detection of potential cancer is mammographic screening, and 25 (of 28) European Union (EU) member states had population-based mammographic screening programmes in 2016 [3].

Sweden’s population-based, organised mammographic screening programme was fully implemented in 1997 [4]. The decision to offer mammographic screening to all 40–74-year-old women, who are nationally registered and have a postal address, was made based on the National Board of Health and Welfare’s recommendations [5] and after considering the pros and cons from existing scientific evidence about an organised mammographic screening programme. At both international and national levels, there is an ongoing debate on the pros and cons of mammographic screening. The benefit of the screening is the reduced rate of mortality from breast cancer. However, the percentages of the reduction in mortality somewhat differ among studies; for instance, one study mentions a 24% decrease in mortality [6], whereas another study reports an estimated 20% decrease [7]. The negative aspects of a mammogram are false-positive results (leading to women being called back for a repeat mammogram although they have no cancer) [7–9], overdiagnosis and subjection to radiation [8]. Since its full implementation in 1997 [4], the Swedish national programme has undergone some adaptations to improve both the participation rates and the programme’s efficiency in relation to the target group. For example, the invitees’ age range was expanded from initially 50–69 (or 50–74 in certain regions in Sweden) [10] to 40–74, uniformly across all regions [5]. Additionally, from charging a patient fee between 0 and 200 SEK (Swedish krona), equivalent to ~ 21 Euro, depending on the women’s region of residence, the programme has been free-of-charge since July 2016 [11].

In Sweden and the other European countries, adjustments and reconsideration will likely occur as technology, societal conditions, state finances, as well as views on what to prioritise change. As a country’s financial conditions influence its healthcare system, as well as other state-funded sectors, priorities must be set on how much resources will be allocated and to what sectors and operations.

In Sweden, as in other countries (such as Belgium, Denmark, Finland and Croatia), the healthcare system is decentralised to some extent [12]. In Swedish, healthcare is a governmental responsibility, but the operational side is delegated to the country’s 21 regions and 290 municipalities [13]. Regional taxes, with some assistance from the government, finance healthcare [14]. The highest organ in each region with the responsibilities of health service delivery, planning and organisation [12] is the Regional Assembly, consisting of directly elected representatives of different Swedish political parties [15]. The Regional Assembly appoints, among others, the Regional Executive Committee, which oversees the finances, as well as coordinates and leads the Regional Assembly’s decisions [16]. Additionally, the Regional Executive Committee may appoint other committees and sub-committees, which in turn are delegated specific responsibilities [17], such as public health and healthcare.

For any screening programme to be efficient and effective, several variables should be considered, such as sustainability, where factors such as political commitments, financial resources and a broad societal understanding of the programme’s pros and cons are important [18]. The participation rate is also an essential factor as a high rate is desirable for a national screening programme to be cost-effective [19] and have a beneficial impact on the population’s health [20]. The Swedish national recommendation for the participation rate in mammographic screening is set at a minimum of 80% [21]. Regional Cancer Centres (RCCs), representing every healthcare region in Sweden [22], recommend different measures to increase the participation rate in mammographic screening. One recommendation is the identification of socioeconomic and/or geographical areas with low participation rates in mammographic screening, and consequently, the analysis of barriers in order to address these in different ways [23]. In the region under study, the participation rate in mammographic screening was 81.4% in 2012, with a 6.8% difference between the municipalities with the highest and the lowest rates [24]. Over the last decade (2009–2019), the highest participation rate in the region was 85% [J. Ramos, Chairman of the National Working Group for Mammography, personal communication, 22 June 2020, unreferenced].

The difference in the participation rates among the municipalities in the region, and with 15–20% of the women declining the invitation to participate in the mammogram, could be a concern as the screening programme is intended to reach as many women as possible for an early detection of potential breast cancer. If the reason for refraining from mammographic screening can be traced back to systematic differences that exclude certain groups in society from participation in the screening, then equity in both health and healthcare is threatened. This issue draws attention to how politicians perceive these differences as they are in the position to make decisions to promote the uptake. To the best of the authors’ knowledge, similar studies regarding regional politicians’ perceptions on mammographic screening have not been published. Additionally, studies concerning participation in mammographic screening have mainly covered Sweden’s three metropolitan areas. This study aims to describe how politicians, representing a region in Sweden, perceive women’s participation in mammographic screening and the politicians’ possibility to promote such participation.

## Materials and methods

Individual semi-structured interviews [25] were conducted, and the data were analysed according to reflexive thematic analysis [26, 27].

### Setting

This study is part of a research project, with the overall focus on accessibility to and participation in mammographic screening in a selected region. Situated in Central Sweden, the chosen region has a population of 273,929 inhabitants, close to the median of 286,547 in Sweden’s 21 regions. The female population comprises 135,888 individuals, also just below the median of 141,947 for this population segment in Sweden’s regions [28]. The region has ten municipalities, and its only mammographic facility is located in the largest municipality, defined as a large city since it has a population of more than 40,000, but less than 200,000 of its residents live in its largest locality [29]. The surrounding municipalities, from where the women have to commute in order to visit the mammographic facility, are situated approximately 20–80 km from it [24]. Due to the self-governance of each region’s healthcare sector in Sweden, a regional perspective is of interest [14].

### Selection of informants

A purposeful sampling was chosen when selecting the informants [30]. All 12 members of a public health and healthcare sub-committee were appointed by the Regional Executive Committee and assigned to further investigate certain healthcare issues, with the power to make decisions concerning resource allocation up to a certain amount and to monitor the region’s work on national agreements and guidelines [17]. The sub-committee members were contacted via email, with a request for their participation in an audiotaped interview regarding mammographic screening.

An information letter about the purpose of the study, with the first author’s (MNvZ) contact details for further information, was attached to the email. This initial invitation was followed up with a telephone call after 14 days. Ten informants (six women and four men) responded to the request; two men neither replied to the email nor answered the telephone call. Reminders were sent out 1 month after the first email. All the informants represented all political parties, except one party, which had no elected representative in this specific sub-committee. The authors did not know any of the informants prior to the commencement of the study.

The informants could choose the interview location. This resulted in eight interviews held in the informants’ respective workplaces, one in the regional university and one over the telephone. The ages of the ten men and women (Informant 1–Informant 10) ranged between 38 and 71 years, and they had served as politicians in the current region for 0.9 to 19 years. For further sociodemographic characteristics of the informants, see Table 1.

**Table 1:** Sociodemographic characteristics of the informants participating to a study on politicians’ perspectives on participation in mammographic screening, Sweden, 2019

Informant	Years active as member of the Regional Executive Committee	Position in the specific Executive Sub-Committee
1	8	(M)
2	6	(M)
3	12	(DM)
4	4	(DM)
5	9	(M)
6	19	(DM)
7	0.9	(M)
8	6	(M)^a^
9	12.8	(M)
10	17	(M)

Notes. Own abbreviations. (M) and (DM) respectively refer to a member and a deputy member of the Executive Sub-Committee

^a^ Denotes the member’s representative in this specific interview

### Data collection

Individual interviews were conducted in Västmanland, 2019, with ten members of a public health and healthcare sub-committee. An interview guide with pre-set but open-ended questions was used [31]. Examples of the questions include the following: “How do you perceive mammographic screening as a diagnostic method to detect potential cancer early?” “What factors do you think could be the reasons why some women refrain from participating in mammographic screening?” “What would you change in order to increase women’s participation in mammographic screening?” (Additional file 1, Interview Guide). The semi-structured interview guide had been tested on other adults, and certain minor modifications of the questions were made for clarification.

All the interviews were conducted by the first author (MNvZ). Before the interviews started, the interviewees were given information about the purpose of the study and their voluntary participation, and a signed informed consent form [32] was obtained from all of them. During each interview, the interviewer made short notes to serve as summary comments on the informant’s responses. After each interview, these notes were verbally read to the informant for verification, offering him/her the opportunity to change, correct or elaborate on his/her answers. Each interview was audiotaped, lasted between 37 and 78 min and was transcribed verbatim by the first author (the transcripts had a 12–35-page range, totalling 214 pages).

### Data analysis

A reflexive thematic analysis with an inductive approach [26, 27] was performed. The analysis already started during the transcription phase, with writing notes from memory. After reading through the transcripts twice, data extracts (text units) relevant to the study’s aim were identified, followed by the coding of the extracts to label what the units contained (Table 2). Next, all codes were gathered, and patterns were sought to gain a deeper understanding of the data and the themes. The process of identifying potential themes included going through the data back and forth to reflect on the themes, sometimes resulting in alterations of the themes. After this phase, the themes were named, with a short definition of each to facilitate the writing of the results. During the whole process, the first author conducted all the steps of the analysis, with the support of the last author, who also reviewed the first author’s interpretation of the data. By doing so, this study’s credibility was strengthened, and the consolidated criteria for reporting qualitative studies (COREQ) were met [33].

**Table 2:** Example of coded transcript by an informant in a study on politicians’ perspectives on participation in mammographic screening, Sweden, 2019

Transcript	Codes
*Interviewer: What would you change to increase the participation rate in mammographic screening?*	
*An informant: I would (…) do more of outreach work. I do believe that I would do more of that, than we do today in such a case.*	More activity Reaching out to women
*Interviewer: Mm, of what have been discussed, what do you perceive as most important and why?*	
*An informant: [Audible exhale, contemplating for a while] Most important and why? Of, of what we have discussed, I believe that (…) the most important is not to be content with 80% or a bit more percentage [participation rate] but to reflect on those who do not participate, and specifically, since we know that in some groups, there is far below 80% who participate, that I think is the important [thing]. And what we can do regarding that.*	Continue to strive Not be content Participation differences among groups Target groups

## Results

The results of the analysis are presented in this section. The themes are illustrated by quotes from the informants. Any words or sentences omitted in the quote are indicated by an ellipsis in parentheses (…). Square brackets containing words or texts of verbal expressions, such as pauses or hesitancies in the response, show that the author [A] has made those clarifications.

The results reflect the informants’ (politicians’) perceptions on both their own and the women’s roles and responsibilities regarding different aspects of participation in mammographic screening. The analysis has identified two main themes: 1) expected actions and 2) expected conditions for acting (see Table 3 for the final themes and sub-themes).

**Table 3:** Main themes with sub-themes in a study on politicians’ perspectives on participation in mammographic screening, Sweden, 2019

Theme	*Sub-themes*
Expected actions	*Obtaining information*
	*Shared commitment to health*
Expected conditions for acting	*Awareness of influential factors*
	*Resources*

In general, the informants (irrespective of party membership) show a high consensus regarding the importance of a high participation rate in mammographic screening. All the informants express their understanding that the responsibility for facilitating participation is shared between the individual and the healthcare system; however, some informants differ slightly in their opinions on the distribution of the responsibility. Additionally, all are aware of the social determinants’ possible impact on the decision to refrain from the screening and that actions can be taken to facilitate participation if there are available resources.

### Theme 1: expected actions

The informants mention that the procedure for participation in mammographic screening is preceded by the action of *obtaining information* from both the invited women and the politicians as decision makers, leading to their preparedness to make a well-informed decision to undergo or refuse the screening (the responsibility of the invited women) or being actively kept informed about the participation rate (the responsibility of the politicians).

#### Obtaining information

The decision to undergo mammographic screening involves an understanding of why a mammogram is conducted and other issues relating to the screening procedure. Some informants perceive this as something that ought to be in a woman’s own interest.

*(…) if you receive an invitation, it ought to be in each and everyone’s interest to find out what it entails.*

According to the informants, the acquisition of information to form a basis of knowledge and understanding is also valid for them as politicians representing the healthcare organisation. For instance, the information about participation rates in mammographic screening is preferably conveyed by communicating with the mammographic unit. One informant states:

*(…) of course, there is a dialogue with the operational units (…). I also believe that they [those responsible for the operational units] best know what works and does not for them. So that is why the politics [politicians] really need to listen to them. Then, you have to make your own judgements, but you have to “take in” and really listen to them [those responsible for the operational units]. It is they who meet the women, in this case. It is they who know. I mean, what, what does one cancel [an appointment for mammographic screening]? Does one cancel or does one just not show up? Or in what way, and who are they? They [the operational units] see this, notice who attends and who are missing. So, one really needs to listen to them as well.*

The preceding quote also highlights the importance of communication with the operational unit.

#### Shared commitment to health

The information and understanding about the procedure, as well as undergoing the screening, are related to a shared commitment to health, in such a way that the informants perceive this for the individual as taking ownership of participating in mammographic screening.

*(…) I believe much in one’s own responsibility. That one has a responsibility for one’s own health in a way. And then, it almost becomes an obligation to go and take that mammogram. That is a bit of how I am thinking. Yes, all these check-ups that one is invited to. Then I think, it is an obligation towards myself. But not everyone thinks so.*

However, the informants do not regard the individual’s duty to take care of one’s health as automatically resulting in participation in mammographic screening. It could mean that a woman conducts a self-examination of her breasts, which improves awareness of her own body.

*Make it a habit to have a routine to conduct [one’s] own breast self-examination (…). The important thing is to get to know one’s own body (…).*

According to the informants (in their role as politicians), their commitment to health concerns an active engagement to strive for improving and facilitating the women’s acquisition of information and awareness of the purpose of the screening, among others.

*I believe that the information from the healthcare may be strengthened (…) so that people do not become scared when they visit the healthcare. To facilitate for them [the individuals] that for instance, [undergoing] a mammogram is not dangerous, to take the screening. Many believe that the machines or that one gets a diagnosis [thrown] in the face, so it is though for many, and many are afraid to receive that information. I believe we [the healthcare organisation] must become better when it comes to information to the patient and take time to talk [with the patient]. Why should I do this [participate in screening], and what does it lead to?*

An occasion to convey the information can be when the women call the mammographic unit for any reason. It could also be done during an event where the politicians meet and greet the residents of a certain suburb or city. These types of events offer the residents the opportunity to ask questions and raise their concerns directly with the politicians. Such events allow more participation than during the actual occasion when the mammogram is performed, which some of the informants consider a quick and standardised procedure compared with a treatment undergone by a person over a period. One way to make participation more than “just taking the mammogram” is to make the woman feel comfortable and safe during the visit, which is a matter of reception. Most of the informants find that increasing the women’s knowledge of the purpose of undergoing mammographic screening is pivotal. Other suggestions to increase the participation rate have been offered, for instance, identifying the groups of women who do not participate by conducting a survey and/or identifying gatekeepers and networks where contacts can be established with these groups of women in order to have a dialogue and/or transfer information regarding screening. Another idea mentioned by some informants is shortening the distance to the mammographic facility, such as via mobile mammographic units that visit the municipalities with less favourable geographic and infrastructural access to the stationary facility. These ideas reflect the politicians’ part in the shared commitment to health, as embedded in the engagement to strive to lower the thresholds for women’s participation in mammographic screening.

### Theme 2: expected conditions for acting

According to the informants’ perceptions, the ability to act on issues concerning participation in mammographic screening requires an *awareness of the influential factors* contributing to the women’s decision to refrain from participating and the *resources* to enable taking action.

#### Awareness of influential factors

The understanding of the impact of social determinants, such as socioeconomic background, on participation in mammographic screening is shared by the informants, as illustrated in this quote:

*(…) we see often that it is certain socioeconomic classes that do not participate in screenings (…) and that ethnic origin (…) also makes a difference (…). If one starts to check who participated [referring to participation rates in 2018], one still notices these differences, that [those with a] higher educational level, (…) they are, they participate (…), more than those with a lower educational level.*

Understanding participation rates at a deeper level than merely the percentages themselves is reflected on, as expressed in this quote:

*(…) it is not just about 20%, but it is that in some groups, we have almost a 100% participation rate, while in some, only 60%. I mean, it is a bigger problem [than [just not having] 80% as one in some groups has a considerably lower [participation rate than 80 %].*

All informants reflect on the fact that some segments of society do not undergo mammographic screening and that it is linked to socioeconomic position, geographic location, ethnicity and culture. If a woman comes from another country, with no previous knowledge of mammographic screening programmes, it is easier for some informants to understand the woman’s decision to refrain from the mammogram, as pointed out by one informant:

*It cannot be due to ignorance that this method [mammographic screening] exists and that it is offered to all women who have turned 40 [years of age]. I do not believe that it is ignorance […]. However, if we look at women who are new [immigrants] in Sweden, then I believe that it can be due to ignorance, cultural, religious reasons why one does not undergo the diagnostics or examination [mammogram].*

All informants mention the impact of low health literacy, which can be due to language barriers and/or low educational level, among others. Some informants reflect on this matter as related to coming from another country, while others mention health literacy more generally.

The social determinants can be viewed in the light of priority setting, which some informants reflect on as vital to understand from a health perspective because these factors have an impact on early diagnosis:

*(…) accessibility is the most important (…) to be able to come and get one’s cancer detected early (…) But besides that, I believe that inequality is the biggest problem. That one clearly can see that there are some groups that cannot partake of this opportunity to the same extent as others.*

#### Resources

The awareness of influential factors is one part of the expected conditions for acting; the other part comprises resources, intangible as well as tangible, in the form of an aspiration, personnel and money. As a resource, an aspiration can be an expression of the importance of participation in mammographic screening, as one informant states:

*Regardless of what political party one belongs to, one agrees that this is an important examination [the mammogram], and something we [the healthcare organisation] shall provide and push for, so women take it.*

When asked about their own initiatives regarding addressing any aspect of participation in mammographic screening, the informants, for different reasons, have not asked any pertinent questions. However, they have discussed matters regarding mammographic screening when it has been brought up by the operational side or other stakeholders during meetings of the sub-committee or the Regional Executive Committee.

If aspiration is one resource, other types of mentioned resources are employees and financial resources and their availability:

*It is about making sure we have the educated personnel who can conduct the examinations [mammograms] and that it also is money in the system to invest in the necessary equipment (…).*

When reflecting on personnel resources, the lack of competent staff to recruit (even though financial resources can be allocated) is brought up as a threat to accessibility and trust in the healthcare organisation, with the risk of undermining public trust in the screening programme itself.

Allocation of resources, purely in monetary terms, is a reality for the informants to consider, as they are responsible for the allocation to the different operational parts of the healthcare organisation.

*Do we allocate more resources to this screening? Then the others [the mammographic unit] have other possibilities to run their operation, of course. So, there we have a great influence (…). They have an assigned mission that is directed by the goal we set, for instance, public health goals (…). Based on that, we set our goals. And then we can locally take directed actions (…) then we can earmark resources (…) if it is really difficult to recruit a specific group of personnel, well then, we have the possibility to give extra salary resources, for example, or whatever one shall call them. So, of course, we can influence.*

Handling the resources involves priority discussions and the possibility to express one’s political will in the budget.

## Discussion

This study has focused on describing how politicians, representing a region in Sweden, perceive women’s participation in mammographic screening, as well as their own possibility, as politicians, to promote such participation. The politicians in this study represent a sub-committee with the responsibility for public health and healthcare issues, and in their role as politicians, they are also representatives of the welfare state and providers of collective resources, such as healthcare [34]. In most cases, resources are finite, and it can be assumed that their allocation and use are not arbitrary. This study’s findings show that resource allocation is preceded by considerations and priority setting, which in turn are based on a combination of an individual’s own perceptions and societal norms, information about mammographic screening as a procedure, understanding the purpose of the mammogram and knowledge of the participation rate in order to observe trends. All are examples of factors that result in some form of decision. For example, the decision might lead to taking action to increase participation rates in mammographic screening but can also result in allocating resources to something else of higher priority. The latter outcome, which can partly be based on values, as well as an understanding of what might maximise health, is supported by other studies’ findings [35, 36].

This study’s main themes concern expected actions and expected conditions for acting, and both include the element of priority. If the state and the healthcare organisation perceive mammographic screening as important for women to undergo or to be maintained as a health service at the population level, it could be assumed that actions to protect this service are vital, such as striving for an even higher participation rate than the current one. This prioritisation should be set by both the women and the politicians and is in fact supported in an official report of the Swedish government, declaring that the state and the individual have a mutual interest in promoting health [37], although their perspectives might differ. The key phrase here is “mutual interest”. Whether one is the invited woman or the elected politician, making health a mutual interest and a priority entails dealing with conflicts of interest and setting priorities in everyday life, which involve actions.

To perceive mammographic screening as highly important, certain requirements can be assumed as essential. Available, evidence-based and understandable information and knowledge form the basis for decisions to be made. Being well informed and committing to strive for health could be viewed from the perspective of priority setting. Is participation in mammographic screening or in other health services, for that matter, important enough for individuals and politicians to prioritise in a reality filled with choices and many other vital issues? Priority setting itself can be based on the estimated severity of a condition. According to a Swedish government bill, three ethical principles should be respected when setting healthcare priorities: human dignity, need and solidarity, and cost-effectivity. Priorities based on needs are as follows (from highest to lowest ranking): life-threatening conditions, prevention and habilitation/rehabilitation, care for less acute and chronic diseases, and care for reasons other than disease or injury [37]. Mammographic screening falls under prevention but is not a condition or a disease in need of treatment, which can be a pitfall as screening may be placed in the interface between health and care. To prevent mammographic screening from being somewhat disregarded, an active engagement to keep updated about participation rates could be assumed as important. The significance of early detection and treatment in contrast to delayed diagnosis and treatment, with the risk of more severe conditions, also needs reflection. A critical reflection on priority setting is called for, as approaches to preventive measures can differ [38]. This issue is addressed in a Swedish report that suggests a national model of priorities in healthcare, reviews the current priority settings and adds more factors to reflect on, such as the severity of the disease that the prevention is meant for [39].

It is vital for all health stakeholders to trust in decisions made on behalf of a country’s citizens and to preserve or gain trust by society at large. Priorities in healthcare require both taking ownership of the responsibility and participation of all health stakeholders [37]. Although the participation may refer to a patient’s right to be actively involved in one’s own health decisions and care, participation also corresponds to the current study’s findings but from a slightly different perspective. The expected actions to be well informed and committed to health require some form of prioritising. Reminders of the purpose of the screening also need to be communicated regularly by the healthcare organisation, as new generations of women are invited, and new technologies may improve the experiences and the procedures surrounding mammographic screening. Additionally, evaluating the initiative to reach out to non-participants of mammographic screening as a step to take action is aligned with rectifying the health inequity [40].

The ability to retrieve and assess information is linked to the social determinants of health [41], in the form of health literacy [42], which is pivotal when making decisions conducive to health and wellbeing. It is also a necessity; as an understanding of the impact of social determinants on health, it becomes an important component of the information and knowledge that politicians must possess in order to act. Without this understanding, the resources, in the form of personnel, finances and an aspiration, might be difficult to mobilise. This study’s results show an awareness of the possible impact of social determinants of health on participation in mammographic screening. This awareness is congruent with previous findings regarding social determinants and participation in screening, such as the level of health literacy, ethnicity, accessibility and socioeconomic position [40, 43–49]. By understanding the impact of social determinants of health, the fundamentals of health equity are also recognised [49]. The informants express their concern that certain social groups display an even lower participation rate than 80%. This is noteworthy as it reflects differences, which can be potential expressions of inequality if the differences are systematic, amendable and unjust [50]. The major goal in Sweden’s public health policies is to create prerequisites for good and equal health; they also express the healthcare organisations’ role in working more towards effective prevention, as well as adjusting healthcare to become more equal by applying proportionate universalism [51]. Proportionate universalism refers to taking action to address health inequalities in society at large but doing so proportionately by considering a gradient where different groups’ conditions result in different needs [52], which is one way to improve equality. However, the informants have not taken the initiative to directly address this situation in relation to mammographic screening, which can indicate the lack of resources, such as financial means, or other priorities taking precedence. It could be of concern as there is a risk of resource allocation in favour of technological equipment, for instance, rather than cancer prevention [53]. This disproportionate resource allocation poses a threat to social equality in health and healthcare, such as offering the population-based mammographic screening service. To increase health equality, a fairly high participation rate is needed [54], and a link to the concept of access becomes plausible since dimensions such as affordability, availability [55] and awareness [56] all relate to this study’s findings.

The findings suggest that a potential dimension could be added to the access account, namely action. As a resource, the aspiration can be embedded in the role of politicians. As they are politicians, they also represent a certain party line; however, their political and ideological stance has not been the objective of this study. Separating the politician from the private person is a difficult, if not an impossible, task. When making any decision, it can be assumed that the person’s set of values on the topic of the decision has an impact. Nonetheless, this set of values may be unconsciously held [57] and may come into awareness when another person (or group) holds another value higher, such as when several actors have to choose among different interventions in which scarce resources have to be invested. In the current study, the informants have reached a consensus regarding the benefits of offering mammographic screening and the importance of having a participation rate that is as high as possible. In turn, this may be explained by the societal norm [57] of the women participating in mammographic screening, which is compatible with the goals valued by the informants, and consciously or unconsciously, influenced by the fact that this health service is offered as a population-based programme endorsed by the government. However, the international and the national debates about the benefits versus the risks of mammographic screening could be assumed to influence the political will to endorse population-based screening. An article about a Swedish debate (published in 1999) presents the argument that the decision to continue the mammographic screening programme should be reconsidered for several reasons, including false-positive results of the mammographic screening (resulting in repeat tests), as well as the insignificant reduction in the mortality rate linked to breast cancer [9]. However, the information provided in the cited paper has been questioned and perceived as misleading in another Swedish article [58]. This is just one example of the different perceptions on the benefits of mammographic screening. For now, the Swedish state considers mammographic screening more beneficial than harmful.

For the politicians, with responsibilities for resource allocation and the possibility to make decisions that may lower the thresholds for people’s access to and participation in a health service, the given information on mammographic screening must account for both benefits and risks of the programme. In this study, many of the informants mention the importance of providing women with information about participating in the screening. This conveying of information will hopefully result in well-informed decisions by the women. However, a well-informed decision requires nuanced information, accounting for both pros and cons of the programme, and this premise is also valid for the politicians. Moreover, being aware and updated about the debate and the scientific evidence regarding mammographic screening can be assumed as expressions of a political commitment since they require engagement. The commitment is an important factor to sustain a cancer screening programme [18] if that is the objective; in turn, this decision should be guided by scientific evidence.

The informants could differ in their perceptions about the appropriate level of the individuals’ own responsibility for their health in relation to the responsibility of the state and might reflect a value base in compliance with each informant’s political orientation. The philosophical discussion on who is responsible for the health could be viewed from different perspectives about the degrees of freedom to act and decide autonomously [59], as well as the division of the responsibility between the individual and society [60]. However, all informants express the need for information and knowledge to make well-informed decisions, the women’s and the state’s (in this case, that of the politicians as proxies for the state) shared commitment to health, and certain requirements for the politicians and the women to perform the expected actions. If all these fundamentals are in place, an activity (as a result of the adopted responsibility) may be undertaken in the form of maintaining or increasing the participation rate in mammographic screening, as well as actively working towards health equity.

### Limitations and strengths

This study concerns the perceptions held by politicians representing a regional sub-committee responsible for issues of public health and healthcare. Since the regions in Sweden are self-governed, the conditions for working on these issues can differ; therefore, the findings may not be transferred to an international or a national context. However, these are for the readers to reflect on and translate the relevance of the findings to their situations and contexts.

Regarding the sampling, the ideal would have been the entire sub-committee’s participation in this study. The selection of the specific sub-committee is perceived as relevant due to the issues that the sub-committee tackles. Other sub-committees and committees in the region have other areas of interest and therefore may not be as updated on the specifics of public health.

The interview guide was not tested on politicians but on other adults, who perceived the questions as easily understandable. Pre-testing the interview guide on individuals who are politicians responsible for healthcare would have been ideal; however, it was difficult to approach politicians with a request for their participation in pre-testing the interview guide. The informants could ask for clarification of the questions at any time during the interviews. However, this was never requested by any informant, and at the end of each interview, the first author summarised the discussion so that the informant could clarify, revise or add some information. These summary comments allowed the informant to verify the interview, contributing to both its accuracy and trustworthiness [61]. Regarding the numbers of interviews and themes, these can be considered small. However, aligned with Braun and Clarke’s [62] approach to conducting reflexive thematic analysis, the sample size should allow patterns in the data to be identified, which the ten interviews in this study have permitted. Regarding the number of themes, none is stipulated, but two or more themes are perceived as sufficient [62].

## Conclusions

This study’s findings indicate that the expected actions and the expected conditions for acting are tightly connected. The expected actions to obtain information and commit to health are based on some form of prioritisation. The understanding of this prioritisation could be traced back to an awareness of influential factors and available resources. Therefore, maintaining or increasing the participation rate in mammographic screening involves a shared responsibility and commitment, favourable conditions and an understanding of mammographic screening as worthwhile to prioritise. Some of the expected conditions for acting (information, knowledge and resources) to make this a priority are common for the potential users of the service, as well as for the politicians. Suggested future research could focus on specific sociodemographic groups with a low participation rate in mammographic screening in order to address potential inequalities in health and healthcare.

## Supplementary Information

**Additional file 1.** Interview guide.

## Data Availability

The reason behind the decision to not make the data publicly available is the protection of the informants’ confidentiality. For any information regarding the transcripts of the interviews or the data analysis, the corresponding author may be of assistance.

## Abbreviations

[A]: Author
COREQ: Consolidated criteria for reporting qualitative studies
DM: Deputy member of the Regional Executive Sub-Committee
M: Member of the Regional Executive Sub-Committee
RCC: Regional Cancer Centre
SEK: Swedish krona
