# Supplementary material for: The politicians’ perspectives on participation in mammographic screening: an interview-based study from a region in Sweden
Source: Arch Public Health. 2021 Apr 17;79:52. doi: 10.1186/s13690-021-00576-6 (PMC8052730; doi:10.1186/s13690-021-00576-6)
Supplement: Supplementary file 1 — Additional file 1. Interview guide. [file 13690_2021_576_MOESM1_ESM.docx]

**Additional File 1. Interview Guide**

The politicians’ perspective on participation in mammographic screening:

an interview-based study from a region in Sweden

Maria Norfjord van Zyl^a^*, Per Tillgren^a^ and Margareta Asp^a^

^a^[School of Health, Care and Social Welfare](about:blank), Mälardalen University, Västerås, Sweden

Corresponding author: Maria Norfjord van Zyl, Division of Public Health Sciences, [School of Health, Care and Social Welfare](about:blank), Mälardalen University, Box 883, 721 23 Västerås, Sweden

E-mail: [maria.norfjord.van.zyl@mdh.se](about:blank)

Per Tillgren, Division of Public Health Sciences, [School of Health, Care and Social Welfare](about:blank), Mälardalen University, Box 883, 721 23 Västerås, Sweden

Margareta Asp, Division of Caring Sciences and Health Care Pedagogics, [School of Health, Care and Social Welfare](about:blank), Mälardalen University, Box 883, 721 23 Västerås, Sweden

**Additional File 1. Interview Guide**

**Opening Question**

Can you tell me about yourself and your experiences in working as a member of the Regional Executive Committee and the sub-committee focusing on public health and healthcare?

**Key Questions**

What do you think about mammographic screening as a diagnostic method to detect potential cancer early?

What do you think are the reasons why some women do not participate in mammographic screening? (What factors can have an impact on their decision to refrain from mammographic screening?)

How do you perceive access to mammographic screening?

How do you perceive participation in mammographic screening?

In your position as a politician, how can you concretely influence access to mammographic screening?

In your position as a politician, how can you concretely influence participation in mammographic screening?

Have you had the opportunity to influence any of the issues that we have touched on above? (If yes, please elaborate.)

**Ending Questions**

Based on what we have discussed, what would you change to increase women’s participation in mammographic screening?

Of what we have discussed, what do you think is the most important issue and why?

**Summary Question***The interviewer presents a summary of what has been said during the interview.*Do you agree with this summary of the interview?

**Final Question**Is there anything you would want to change or add?
